# *N*-{4-[(3-Methyl­phen­yl)sulfamo­yl]phen­yl}acetamide

**DOI:** 10.1107/S1600536812001274

**Published:** 2012-01-18

**Authors:** Muhammad Akhyar Farrukh, Fahim Ashraf Qureshi, Ahmad Adnan, Sevim Türktekin, Mehmet Akkurt

**Affiliations:** a Department of Chemistry, Government College University, Lahore 54000, Pakistan; b Department of Physics, Faculty of Sciences, Erciyes University, 38039 Kayseri, Turkey

## Abstract

In the title compound, C_15_H_16_N_2_O_3_S, the central C—S(=O)_2_N(H)—C unit is twisted, with a C—S—N—C torsion angle of −56.4 (2)°. The benzene rings form a dihedral angle of 49.65 (15)° with each other. In the crystal, mol­ecules are linked by N—H⋯O hydrogen bonds, generating a three-dimensional network.

## Related literature

For background to sulfonamides, see: Ahmad *et al.* (2011*a* ▶,*b* ▶); Faryal *et al.* (2011 ▶); Pandya *et al.* (2003 ▶); Singh & Bansal (2004 ▶). For the crystal structure of the isomeric compound, *N*–{4–[(4–methyl­phen­yl)sulfamo­yl]phen­yl}acetamide, see: John *et al.* (2010 ▶).
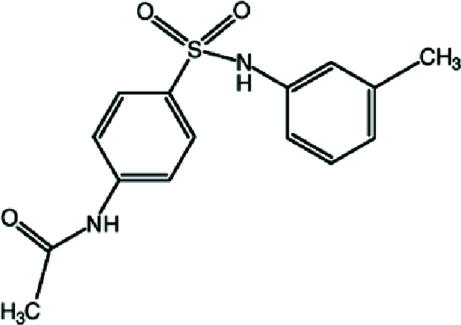


## Experimental

### 

#### Crystal data

C_15_H_16_N_2_O_3_S
*M* *_r_* = 304.37
Orthorhombic, 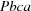

*a* = 12.4072 (4) Å
*b* = 9.8528 (4) Å
*c* = 24.7872 (10) Å
*V* = 3030.1 (2) Å^3^
*Z* = 8
Mo *K*α radiation
μ = 0.23 mm^−1^
*T* = 296 K
0.13 × 0.10 × 0.05 mm

#### Data collection

Bruker APEXII CCD diffractometer
27585 measured reflections
3766 independent reflections
2018 reflections with *I* > 2σ(*I*)
*R*_int_ = 0.089

#### Refinement

*R*[*F* ^2^ > 2σ(*F* ^2^)] = 0.059
*wR*(*F* ^2^) = 0.146
*S* = 1.02
3766 reflections
200 parameters
2 restraints
H atoms treated by a mixture of independent and constrained refinement
Δρ_max_ = 0.44 e Å^−3^
Δρ_min_ = −0.34 e Å^−3^

### 

Data collection: *APEX2* (Bruker, 2007 ▶); cell refinement: *SAINT* (Bruker, 2007 ▶); data reduction: *SAINT*; program(s) used to solve structure: *SHELXS97* (Sheldrick, 2008 ▶); program(s) used to refine structure: *SHELXL97* (Sheldrick, 2008 ▶); molecular graphics: *ORTEP-3 for Windows* (Farrugia, 1997 ▶); software used to prepare material for publication: *WinGX* (Farrugia, 1999 ▶) and *PLATON* (Spek, 2009 ▶).

## Supplementary Material

Crystal structure: contains datablock(s) global, I. DOI: 10.1107/S1600536812001274/lx2224sup1.cif

Structure factors: contains datablock(s) I. DOI: 10.1107/S1600536812001274/lx2224Isup2.hkl

Supplementary material file. DOI: 10.1107/S1600536812001274/lx2224Isup3.cml

Additional supplementary materials:  crystallographic information; 3D view; checkCIF report

## Figures and Tables

**Table 1:** Hydrogen-bond geometry (Å, °)

*D*—H⋯*A*	*D*—H	H⋯*A*	*D*⋯*A*	*D*—H⋯*A*
N1—H1*N*⋯O2^i^	0.86 (2)	2.09 (2)	2.938 (3)	171 (2)
N2—H2*N*⋯O3^ii^	0.84 (2)	2.05 (2)	2.878 (3)	173 (3)

Symmetry codes: (i) 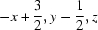; (ii) 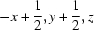.

**Fig. 1.**
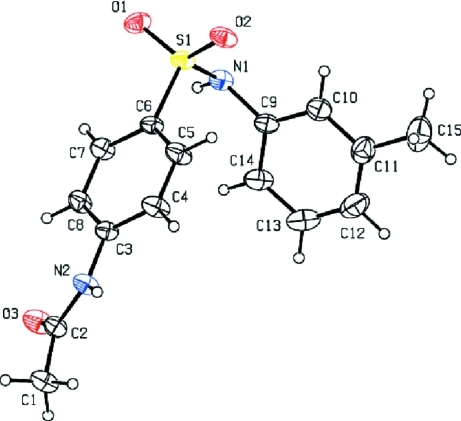
The molecular structure of the title compound with the atom numbering scheme. Displacement ellipsoids are drawn at the 30% probability level. H atoms are presented as small spheres of arbitrary radius.

**Fig. 2.**
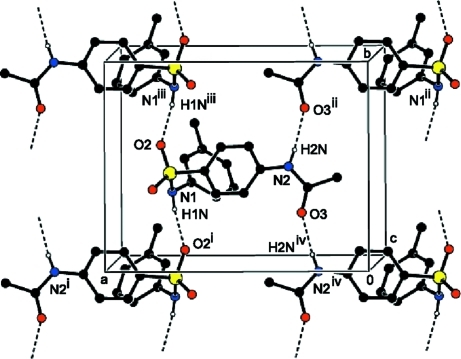
A view of the N—H···O interactions (dotted lines) in the crystal structure of the title compound. H atoms non–participating in hydrogen- bonding were omitted for clarity. [Symmetry codes: (i) - x + 3/2, y - 1/2, z; (ii) - x + 1/2, y + 1/2, z; (iii) - x + 3/2, y + 1/2, z; (iv) - x + 1/2, y - 1/2, z.]

## supplementary crystallographic information

### Comment

Sulfonamides are a diverse group of compounds having considerable medical importance (Pandya *et al.*, 2003). They are the very important class of compounds in the pharmaceutical industry, being widely used as anticancer, anti-inflammatory and antiviral agents. They have been the center of drug structures as this group is quite stable and well tolerated in human beings (Singh & Bansal 2004). Compounds bearing sulfonate group increases their hydrophilicity and have become useful pharmacological tool. In continuation of on–going structural studies of sulfonamide derivatives (Faryal *et al.*, 2011; Ahmad *et al.*, 2011*a*,*b*), the crystal structure of title sulfonamide *N*–{4–[(3–methylphenyl)sulfamoyl]phenyl}acetamide is described herein.

The title compound, (Fig. 1) is a isomer of the compound, *N*–{4–[(4–methylphenyl)sulfamoyl]phenyl}acetamide, reported by John *et al.* (2010). The title compound crystallizes in the orthorhombic space group *P* bca with *Z*=8, while its mentioned-isomer crystallizes in triclinic space group P-1 with *Z*=2. The values of the geometric properties of both compounds are similar. The dihedral angle between the C3–C8 and C9–C14 benzene rings is 49.65 (15) °. In the central C—S(═O)_2_N(H)—C unit of title compound, the C6—S1—N1—C9 torsion angle of -56.4 (2)° indicates a twist in the molecule. The amide group is not co–planar with the benzene ring to which it is attached [C2—N2—C3—C8 = 27.5 (5) °]. In the crystal structure, molecules are connected *via* N—H···O hydrogen bonds, generating a three–dimensional network (Table 1, Fig. 2).

### Experimental

5 mmol of *m*–toluidine was dissolved in 20 ml of distilled water then 5 mmol of 4–acetamidobenzenesulfonyl chloride was added. The reaction mixture was stirred for about 2–3 h while the pH of the reaction mixture was maintained between 8–10 using 3% Na_2_CO_3_. The reaction was monitored by TLC. The precipitate formed was filtered, washed with distilled water, dried and recrystallized by using methanol.

### Refinement

The NH H–atoms were located in a difference Fourier map. They were isotropically refined with a distance restraint: N—H = 0.86 (2) Å. The C–bound H–atoms were positioned geometrically [C—H = 0.93 and 0.96 Å., for aromatic and methyl H–atoms, respectively], and constrained to ride on their parent atoms, with *U*_iso_=1.2*U*_eq_ (C_aromatic_) and *U*_iso_=1.5*U*_eq_ (C_methyl_). In the final refinement one low angle reflection evidently effected by the beam stop was omitted, *i.e.* 0 0 2.

### Crystal data

**Table:**

C_15_H_16_N_2_O_3_S	*F*(000) = 1280
*M**_r_* = 304.37	*D*_x_ = 1.334 Mg m^−^^3^
Orthorhombic, *P**b**c**a*	Mo *K*α radiation, λ = 0.71073 Å
Hall symbol: -P 2ac 2ab	Cell parameters from 2598 reflections
*a* = 12.4072 (4) Å	θ = 2.8–26.1°
*b* = 9.8528 (4) Å	µ = 0.23 mm^−^^1^
*c* = 24.7872 (10) Å	*T* = 296 K
*V* = 3030.1 (2) Å^3^	Plates, colourless
*Z* = 8	0.13 × 0.10 × 0.05 mm

### Data collection

**Table:**

Bruker APEXII CCD diffractometer	2018 reflections with *I* > 2σ(*I*)
Radiation source: sealed tube	*R*_int_ = 0.089
graphite	θ_max_ = 28.3°, θ_min_ = 2.8°
φ and ω scans	*h* = −16→15
27585 measured reflections	*k* = −13→13
3766 independent reflections	*l* = −32→32

### Refinement

**Table:**

Refinement on *F*^2^	Primary atom site location: structure-invariant direct methods
Least-squares matrix: full	Secondary atom site location: difference Fourier map
*R*[*F*^2^ > 2σ(*F*^2^)] = 0.059	Hydrogen site location: difference Fourier map
*wR*(*F*^2^) = 0.146	H atoms treated by a mixture of independent and constrained refinement
*S* = 1.02	*w* = 1/[σ^2^(*F*_o_^2^) + (0.0559*P*)^2^ + 1.2606*P*] where *P* = (*F*_o_^2^ + 2*F*_c_^2^)/3
3766 reflections	(Δ/σ)_max_ = 0.001
200 parameters	Δρ_max_ = 0.44 e Å^−^^3^
2 restraints	Δρ_min_ = −0.33 e Å^−^^3^

### Special details

**Table:**

Geometry. Bond distances, angles etc. have been calculated using the rounded fractional coordinates. All su's are estimated from the variances of the (full) variance-covariance matrix. The cell esds are taken into account in the estimation of distances, angles and torsion angles
Refinement. Refinement on F^2^ for ALL reflections except those flagged by the user for potential systematic errors. Weighted R-factors wR and all goodnesses of fit S are based on F^2^, conventional R-factors R are based on F, with F set to zero for negative F^2^. The observed criterion of F^2^ > 2sigma(F^2^) is used only for calculating -R-factor-obs etc. and is not relevant to the choice of reflections for refinement. R-factors based on F^2^ are statistically about twice as large as those based on F, and R-factors based on ALL data will be even larger.

### Fractional atomic coordinates and isotropic or equivalent isotropic displacement parameters (Å^2^)

**Table:**

	*x*	*y*	*z*	*U*_iso_*/*U*_eq_	
S1	0.75972 (5)	0.46076 (6)	0.12164 (3)	0.0386 (2)	
O1	0.82358 (15)	0.3853 (2)	0.08468 (9)	0.0510 (7)	
O2	0.79502 (15)	0.59357 (18)	0.13737 (9)	0.0477 (7)	
O3	0.25638 (16)	0.27762 (18)	0.04342 (10)	0.0573 (8)	
N1	0.75422 (19)	0.3683 (2)	0.17624 (10)	0.0407 (8)	
N2	0.30625 (19)	0.4969 (2)	0.05052 (11)	0.0424 (8)	
C1	0.1211 (2)	0.4459 (3)	0.03050 (14)	0.0536 (10)	
C2	0.2336 (2)	0.3979 (3)	0.04182 (11)	0.0400 (9)	
C3	0.4142 (2)	0.4837 (2)	0.06584 (11)	0.0362 (9)	
C4	0.4594 (2)	0.5905 (3)	0.09375 (13)	0.0505 (12)	
C5	0.5659 (2)	0.5860 (3)	0.10976 (13)	0.0501 (10)	
C6	0.6270 (2)	0.4738 (3)	0.09778 (11)	0.0362 (9)	
C7	0.5827 (2)	0.3668 (3)	0.06968 (12)	0.0448 (10)	
C8	0.4762 (2)	0.3707 (3)	0.05379 (12)	0.0442 (10)	
C9	0.6853 (2)	0.4104 (3)	0.21957 (12)	0.0425 (10)	
C10	0.7132 (2)	0.5202 (3)	0.25144 (13)	0.0494 (11)	
C11	0.6485 (3)	0.5624 (4)	0.29349 (13)	0.0593 (12)	
C12	0.5563 (3)	0.4898 (4)	0.30367 (16)	0.0723 (16)	
C13	0.5285 (3)	0.3793 (4)	0.27305 (18)	0.0780 (16)	
C14	0.5919 (2)	0.3389 (3)	0.23047 (15)	0.0596 (13)	
C15	0.6791 (4)	0.6837 (4)	0.32666 (16)	0.0900 (19)	
H1A	0.10860	0.44510	−0.00770	0.0800*	
H1B	0.11240	0.53650	0.04400	0.0800*	
H1C	0.07050	0.38670	0.04790	0.0800*	
H1N	0.746 (2)	0.2849 (18)	0.1672 (10)	0.034 (7)*	
H2N	0.283 (2)	0.5766 (19)	0.0497 (11)	0.041 (8)*	
H4	0.41780	0.66630	0.10190	0.0610*	
H5	0.59610	0.65850	0.12850	0.0600*	
H7	0.62490	0.29160	0.06140	0.0540*	
H8	0.44620	0.29800	0.03510	0.0530*	
H10	0.77690	0.56660	0.24440	0.0590*	
H12	0.51180	0.51610	0.33200	0.0870*	
H13	0.46600	0.33130	0.28110	0.0930*	
H14	0.57240	0.26480	0.20940	0.0710*	
H15A	0.65440	0.76480	0.30910	0.1350*	
H15B	0.75610	0.68710	0.33050	0.1350*	
H15C	0.64650	0.67700	0.36170	0.1350*	

### Atomic displacement parameters (Å^2^)

**Table:**

	*U*^11^	*U*^22^	*U*^33^	*U*^12^	*U*^13^	*U*^23^
S1	0.0279 (3)	0.0296 (3)	0.0582 (5)	0.0008 (3)	0.0013 (3)	−0.0014 (3)
O1	0.0385 (11)	0.0465 (12)	0.0679 (14)	0.0097 (10)	0.0113 (10)	−0.0046 (10)
O2	0.0364 (10)	0.0307 (10)	0.0759 (15)	−0.0076 (9)	−0.0033 (10)	−0.0003 (10)
O3	0.0501 (13)	0.0258 (11)	0.0960 (18)	−0.0051 (10)	−0.0081 (12)	0.0021 (10)
N1	0.0356 (13)	0.0279 (12)	0.0586 (16)	0.0013 (11)	0.0000 (12)	−0.0011 (11)
N2	0.0339 (13)	0.0240 (12)	0.0694 (17)	0.0010 (10)	−0.0097 (12)	0.0005 (11)
C1	0.0382 (16)	0.0475 (18)	0.075 (2)	−0.0051 (15)	−0.0110 (16)	0.0012 (16)
C2	0.0397 (16)	0.0319 (15)	0.0485 (18)	−0.0043 (13)	−0.0039 (13)	0.0016 (13)
C3	0.0316 (15)	0.0260 (13)	0.0509 (18)	−0.0009 (12)	−0.0003 (13)	0.0017 (12)
C4	0.0342 (16)	0.0254 (14)	0.092 (3)	0.0053 (13)	−0.0061 (16)	−0.0113 (15)
C5	0.0397 (16)	0.0277 (14)	0.083 (2)	−0.0017 (13)	−0.0079 (15)	−0.0133 (14)
C6	0.0267 (13)	0.0298 (14)	0.0521 (18)	0.0010 (12)	0.0003 (12)	0.0001 (12)
C7	0.0384 (17)	0.0301 (14)	0.066 (2)	0.0063 (13)	−0.0031 (15)	−0.0093 (13)
C8	0.0412 (16)	0.0304 (14)	0.061 (2)	0.0004 (13)	−0.0070 (14)	−0.0103 (13)
C9	0.0302 (15)	0.0436 (16)	0.0537 (19)	0.0049 (13)	−0.0024 (13)	0.0037 (14)
C10	0.0444 (18)	0.0487 (18)	0.055 (2)	0.0035 (15)	−0.0033 (15)	0.0024 (16)
C11	0.062 (2)	0.066 (2)	0.050 (2)	0.0162 (19)	−0.0014 (17)	−0.0035 (17)
C12	0.058 (2)	0.096 (3)	0.063 (3)	0.015 (2)	0.0167 (19)	0.002 (2)
C13	0.045 (2)	0.091 (3)	0.098 (3)	−0.004 (2)	0.019 (2)	0.006 (3)
C14	0.0399 (18)	0.058 (2)	0.081 (3)	−0.0041 (16)	0.0054 (18)	−0.0009 (18)
C15	0.107 (4)	0.100 (3)	0.063 (3)	0.016 (3)	−0.001 (2)	−0.022 (2)

### Geometric parameters (Å, °)

**Table:**

S1—O1	1.421 (2)	C10—C11	1.380 (5)
S1—O2	1.4339 (19)	C11—C12	1.373 (5)
S1—N1	1.633 (2)	C11—C15	1.500 (5)
S1—C6	1.754 (3)	C12—C13	1.371 (6)
O3—C2	1.219 (3)	C13—C14	1.375 (5)
N1—C9	1.434 (4)	C1—H1A	0.9600
N2—C2	1.346 (4)	C1—H1B	0.9600
N2—C3	1.398 (3)	C1—H1C	0.9600
N1—H1N	0.858 (18)	C4—H4	0.9300
N2—H2N	0.84 (2)	C5—H5	0.9300
C1—C2	1.500 (4)	C7—H7	0.9300
C3—C8	1.386 (4)	C8—H8	0.9300
C3—C4	1.379 (4)	C10—H10	0.9300
C4—C5	1.380 (4)	C12—H12	0.9300
C5—C6	1.373 (4)	C13—H13	0.9300
C6—C7	1.378 (4)	C14—H14	0.9300
C7—C8	1.379 (4)	C15—H15A	0.9600
C9—C14	1.383 (4)	C15—H15B	0.9600
C9—C10	1.384 (4)	C15—H15C	0.9600
			
O1—S1—O2	118.84 (12)	C11—C12—C13	121.4 (4)
O1—S1—N1	105.41 (12)	C12—C13—C14	120.7 (3)
O1—S1—C6	110.14 (13)	C9—C14—C13	118.8 (3)
O2—S1—N1	107.25 (12)	C2—C1—H1A	109.00
O2—S1—C6	108.15 (13)	C2—C1—H1B	109.00
N1—S1—C6	106.32 (13)	C2—C1—H1C	109.00
S1—N1—C9	118.97 (18)	H1A—C1—H1B	109.00
C2—N2—C3	128.2 (2)	H1A—C1—H1C	109.00
S1—N1—H1N	108.8 (17)	H1B—C1—H1C	110.00
C9—N1—H1N	113.7 (17)	C3—C4—H4	120.00
C2—N2—H2N	116.5 (17)	C5—C4—H4	120.00
C3—N2—H2N	115.1 (17)	C4—C5—H5	120.00
O3—C2—N2	123.0 (2)	C6—C5—H5	120.00
O3—C2—C1	121.9 (2)	C6—C7—H7	120.00
N2—C2—C1	115.1 (2)	C8—C7—H7	120.00
N2—C3—C4	117.1 (2)	C3—C8—H8	120.00
C4—C3—C8	119.7 (2)	C7—C8—H8	120.00
N2—C3—C8	123.2 (2)	C9—C10—H10	119.00
C3—C4—C5	120.6 (3)	C11—C10—H10	119.00
C4—C5—C6	119.5 (3)	C11—C12—H12	119.00
C5—C6—C7	120.3 (2)	C13—C12—H12	119.00
S1—C6—C5	120.3 (2)	C12—C13—H13	120.00
S1—C6—C7	119.3 (2)	C14—C13—H13	120.00
C6—C7—C8	120.3 (3)	C9—C14—H14	121.00
C3—C8—C7	119.5 (3)	C13—C14—H14	121.00
N1—C9—C14	119.9 (3)	C11—C15—H15A	110.00
C10—C9—C14	119.8 (3)	C11—C15—H15B	110.00
N1—C9—C10	120.3 (2)	C11—C15—H15C	110.00
C9—C10—C11	121.4 (3)	H15A—C15—H15B	109.00
C10—C11—C12	117.8 (3)	H15A—C15—H15C	109.00
C10—C11—C15	120.5 (3)	H15B—C15—H15C	109.00
C12—C11—C15	121.7 (3)		
			
O1—S1—N1—C9	−173.3 (2)	C8—C3—C4—C5	0.1 (4)
O2—S1—N1—C9	59.1 (2)	C3—C4—C5—C6	−0.1 (5)
C6—S1—N1—C9	−56.4 (2)	C4—C5—C6—C7	0.4 (4)
O2—S1—C6—C7	166.2 (2)	C4—C5—C6—S1	−175.7 (2)
N1—S1—C6—C7	−78.9 (2)	S1—C6—C7—C8	175.5 (2)
O2—S1—C6—C5	−17.6 (3)	C5—C6—C7—C8	−0.7 (4)
N1—S1—C6—C5	97.3 (3)	C6—C7—C8—C3	0.6 (4)
O1—S1—C6—C5	−149.0 (2)	N1—C9—C10—C11	−179.8 (3)
O1—S1—C6—C7	34.9 (3)	C14—C9—C10—C11	−1.4 (5)
S1—N1—C9—C10	−73.2 (3)	N1—C9—C14—C13	178.4 (3)
S1—N1—C9—C14	108.4 (3)	C10—C9—C14—C13	0.1 (5)
C2—N2—C3—C4	−153.2 (3)	C9—C10—C11—C12	1.5 (5)
C3—N2—C2—O3	−4.5 (5)	C9—C10—C11—C15	−178.6 (3)
C2—N2—C3—C8	27.5 (5)	C10—C11—C12—C13	−0.4 (6)
C3—N2—C2—C1	174.8 (3)	C15—C11—C12—C13	179.7 (4)
N2—C3—C4—C5	−179.2 (3)	C11—C12—C13—C14	−0.9 (6)
C4—C3—C8—C7	−0.3 (4)	C12—C13—C14—C9	1.1 (5)
N2—C3—C8—C7	179.0 (3)		

### Hydrogen-bond geometry (Å, °)

**Table:**

*D*—H···*A*	*D*—H	H···*A*	*D*···*A*	*D*—H···*A*
N1—H1N···O2^i^	0.86 (2)	2.09 (2)	2.938 (3)	171 (2)
N2—H2N···O3^ii^	0.84 (2)	2.05 (2)	2.878 (3)	173 (3)

Symmetry codes: (i) −*x*+3/2, *y*−1/2, *z*; (ii) −*x*+1/2, *y*+1/2, *z*.
